# Tyrosinase-Targeting Gallacetophenone Inhibits Melanogenesis in Melanocytes and Human Skin- Equivalents

**DOI:** 10.3390/ijms21093144

**Published:** 2020-04-29

**Authors:** Ji Young Lee, Jooyun Lee, Daejin Min, Juewon Kim, Hyoung-June Kim, Kyoung Tai No

**Affiliations:** 1 Amorepacific Corporation R&D Center, Yongin City, Gyunggi-do 17074, Korea; jy_lee@amorepacific.com (J.Y.L.); djmin@amorepacific.com (D.M.); jwkim@amorepacific.com (J.K.); 2 Department of Bioengineering, Yonsei University, Seoul 03722, Korea; 3 Bioinformatics and Molecular Design Research Center (BMDRC), Yonsei University, Incheon 21983, Korea; bbjylee@gmail.com

**Keywords:** tyrosinase, homology modeling, virtual screening, natural compound, gallacetophenone, human skin equivalent

## Abstract

Demands for safe depigmentation compounds are constantly increasing in the pharmaceutical and cosmetic industry, since the numerous relevant compounds reported to date have shown undesirable side effects or low anti-melanogenic effects. In this study, we reported three novel inhibitors of tyrosinase, which is the key enzyme in melanogenesis, identified using docking-based high throughput virtual screening of an in-house natural compound library followed by mushroom tyrosinase inhibition assay. Of the three compounds, gallacetophenone showed high anti-melanogenic effect in both human epidermal melanocytes and a 3D human skin model, MelanoDerm. The inhibitory effect of gallacetophenone on tyrosinase was elucidated by computational molecular modeling at the atomic level. Binding of gallacetophenone to the active site of tyrosinase was found to be stabilized by hydrophobic interactions with His367, Ile368, and Val377; hydrogen bonding with Ser380 and a water molecule bridging the copper ions. Thus, our results strongly suggested gallacetophenone as an anti-melanogenic ingredient that inhibits tyrosinase.

## 1. Introduction

Melanin, a cluster of natural pigments, protects the skin by absorbing the harmful ultraviolet radiation (UVR) [1]. However, abnormal accumulation of melanin in the skin results in dermatological problems such as freckles, lentigo, age spots, and melisma [2,3,4,5]. Since melanin is synthesized via a series of enzymatic reactions called melanogenesis, controlling the latter may revert the process as desired and lead to the identification of effective skin-whitening compounds for medicines and cosmetics [6,7,8,9].

Melanogenesis is a process involving the catalysis of tyrosine by tyrosinase, tyrosinase-related protein 1 (TRP1), tyrosinase-related protein 2 (TRP2)/dopachrome tautomerase (DCT), and microphthalmia-associated transcription factor (MITF) [10,11]. In melanogenesis, MITF is a major transcription factor that regulates melanogenic genes encoding tyrosinase and TRP2/DCT [12,13]. Tyrosinase is the rate-limiting enzyme catalyzing tyrosine, and TRP1 is responsible for the oxidation of 5,6-dihydroxyindole-2-carboxylic acid to a carboxylated indole quinone [14]. TRP2/DCT catalyze dopachrome to 5,6-dihydroxyindole-2-carboxylic acid [15].

Melanins include eumelanin (brown/black) and pheomelanin (yellow/red) and are produced from L-tyrosine. The initial step of melanin synthesis involves hydroxylation of L-tyrosine to dihydroxyphenylalanine (DOPA), and subsequently oxidation of DOPA to DOPA-quinone by tyrosinase [16,17,18,19,20], with DOPA-quinone eventually being oxidized to dopachrome. Tyrosinase catalyzes dopachrome to 5,6-dihydroxyindole (DHI). Eumelanin is produced from a series of reactions between DHI and dihydroxyindole-2-carboxlic acid (DHICA) [10,11,21,22], while pheomelanin is produced by the condensation of dopaquinone with cysteine or glutathione into cysteinyl dopa or glutathionyl dopa [23,24]. In the process of melanogenesis, the rate-limiting enzyme, tyrosinase, is the key enzyme that initiates the process and catalyzes two subsequent reactions. Therefore, inhibition of tyrosinase might be one of the effective ways to achieve a whitening effect or treat hyperpigmentation.

Till date, various inhibitors of melanogenesis-related enzymes have been identified for whitening effects and the control of skin pigmentation in the medicine and cosmetic industry [10,25,26]. Unfortunately, these inhibitors have undesirable side effects, owing to which the demand for alternative anti-melanogenic compounds has been increasing. Hydroquinone, which is one of the best-known clinical depigmentation agents, is known to cause erythema, stinging, irritation, and allergic contact dermatitis [16]. Even kojic acid (KA), which is an ingredient commonly used in cosmetic products, has several side effects, causing genotoxic, hepatocarcinogenic, and allergic dermatitis [16,27].

Therefore, novel compounds with anti-melanogenic activity and overall safety are considered necessary. In this study, we screened an in-house natural compound library for safe and effective compounds [17].

The purpose of this work was to identify new natural compounds with anti-melanogenic activity. We generated a human tyrosinase structure and performed docking-based high throughput virtual screening. Finally, we screened three effective hit compounds, out of a total of 74. Of the three, a novel tyrosinase inhibitor, 1-(2,3,4-trihydroxyphenyl)ethanone (gallacetophenone), was examined for anti-melanogenic effect in human epidermal melanocytes and a human skin equivalent, besides performing a computational molecular modeling. Based on our findings, we suggest gallacetophenone as a novel anti-melanogenic compound with a potential to inhibit tyrosinase activity.

## 2. Results

### 2.1. Homology Modeling of Human Tyrosinase and Docking-Based High Throughput Virtual Screening

**Figure 1:**
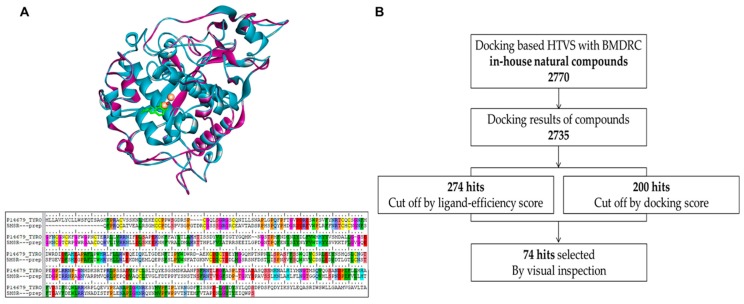
Homology modeling of human tyrosinase and docking-based high throughput virtual screening (**A**) Superimposed protein structures (top) and aligned protein sequences (bottom) of human tyrosinase model (cyan) and a human TRP1 protein template (PDB 5M8R: purple): the copper ions and co-crystalized ligands (including a water molecule) are represented by spheres (salmon) and sticks (green), respectively. (**B**) The schematic diagram shows the process of docking-based high throughput virtual screening (HTVS).

To generate the 3D structure of human tyrosinase, we performed homology modeling using Small-Molecule Drug Discovery Suite 2018-3 (Schrödinger, NY, USA). The full sequence of human tyrosinase (P14679) was acquired from the UniProt database [28], and the X-ray crystal structure of human tyrosinase-related protein 1 (TRP1, PDB ID: 5M8R) [29] was used as a protein template, considering its 42.6% sequence identity with human tyrosinase. The co-crystallized ligand, copper ions instead of zinc ions, and a water molecule bridging the two metal ions were reflected in the human tyrosinase model. The Protein Preparation Wizard (Schrödinger, NY, USA) was used to assign bond orders and charge states to ionizable residues and to perform a restrained minimization of human tyrosinase model. Low-energy 3D structures of compounds in the in-house natural compound library were generated by LigPrep and docked into the active site of human tyrosinase model using Glide in the Standard Precision mode, based on a grid box of 20 × 20 × 20 Å^3^ centered on the co-crystallized ligand. A structural superimposition of the human tyrosinase model and TRP1, as well as the aligned protein sequence, are shown in Figure 1A. Compounds with no binding mode were filtered out. The docking results of 2735 compounds were ranked and filtered by docking score and ligand-efficiency score; finally, 74 compounds with satisfactory hydrophobic interactions with His367, Ile368, and Val377; hydrogen bonding with Ser380; and a water molecule bridging the copper ions were selected by visual inspection. The process of docking-based high throughput virtual screening is shown in Figure 1B.

### 2.2. Compounds Selected from Docking-Based High Throughput Virtual Screening Inhibited Mushroom Tyrosinase

**Figure 2:**
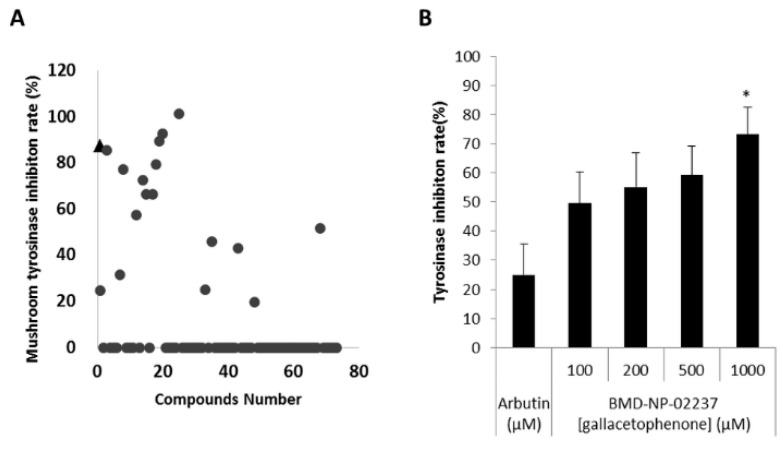

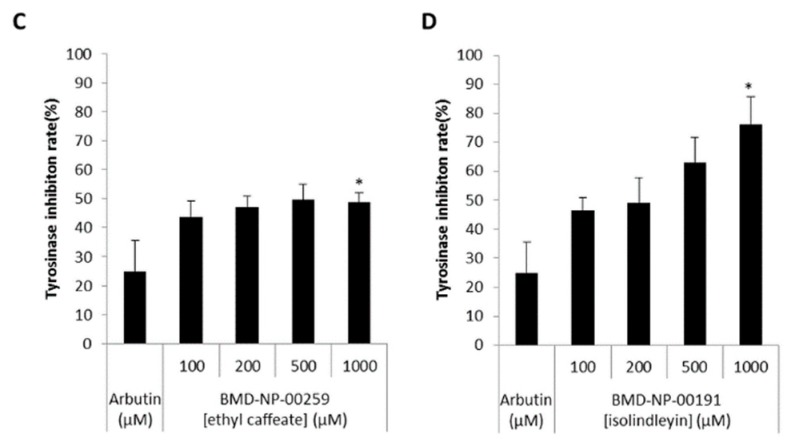
Mushroom tyrosinase inhibition rate using screened hit compounds. (**A**) A total of 74 compounds from docking-based high throughput virtual screening are represented in the graph in terms of mushroom tyrosinase inhibition rate. Arbutin, a positive control, is represented as a triangle (▲), while the other compounds are represented as circles (●). Of the screened compounds, three were selected for another round of mushroom tyrosinase inhibition assay in a dose-dependent manner. The mushroom tyrosinase inhibition rate increased under treatment with (**B**) BMD-NP-02237(Gallacetophenone); (**C**) BMD-NP-00259(Ethyl caffeate); and (**D**) BMD-NP-00191(Isolindleyin). Arbutin was treated at a concentration of 500 μM. The assay results are normalized as per the manufacturer’s instruction. Data are expressed as the mean ± SD of at least three independent measurements (*p < 0.05).

A mushroom tyrosinase inhibitor screening assay was conducted to confirm the inhibitory effect of the 74 hit compounds obtained through docking-based high throughput virtual screening (Figure 2A). Four of these compounds showed > 80% inhibition rate, with arbutin as a positive control (Figure S1). Of the four compounds, BMD-NP-01814 (Glabridin) has been studied extensively in relation to whitening [30,31]. Excluding glabridin, inhibition rates of the remaining three hit compounds were measured with indicated concentrations. As shown in Figure 2B,D, BMD-NT-02237 (Gallacetophenone) and BMD-NT-00191 (Isolindleyin) clearly inhibited mushroom tyrosinase in a dose-dependent manner. However, BMD-NT-00259 (ethyl caffeate) inhibited mushroom tyrosinase to a slightly lesser extent than the other two (Figure 2C). The structures of the four compounds and arbutin are shown in Figure S2.

### 2.3. Gallacetophenone Decreased Melanin Content of Human Epidermal Melanocytes

**Figure 3:**
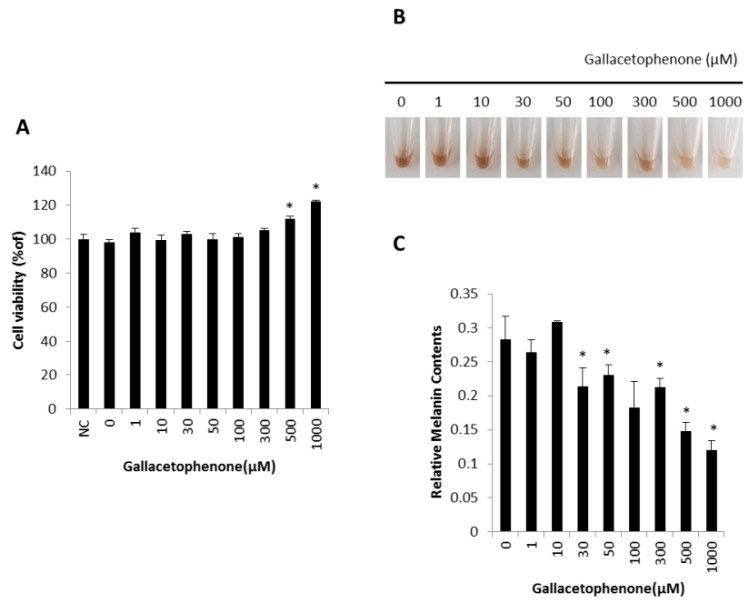
Anti-melanogenic effect of gallacetophenone in human epidermal melanocytes. (**A**) Cell viability after treatment with various concentrations of gallacetophenone; (**B**) the color of cell lysate; (**C**) the melanin content determined using cell lysates. Data are expressed as the mean ± SD of at least three independent measurements (*p < 0.05).

Next, we investigated whether gallacetophenone had anti-melanogenic effect in human epidermal melanocytes. Gallacetophenone showed the highest inhibition rate in the mushroom tyrosinase inhibitor screening assay (Figure S1) and exhibited significant inhibition in a dose-dependent manner (Figure 2B). To confirm the function of gallacetophenone in human epidermal melanocytes, a toxicity assay of gallacetophenone was performed first. As shown in Figure 3A, gallacetophenone did not show cytotoxicity at concentrations up to 1000 μM in human epidermal melanocytes. Based on the cytotoxicity data, human epidermal melanocytes were treated with various concentrations of gallacetophenone for 7 days. The color of cell lysate in gallacetophenone-treated cells became lighter in a dose-dependent manner (Figure 3B), and the melanin content was significantly decreased (Figure 3C).

### 2.4. Whitening Effect of Gallacetophenone Was Observed in 3D Human Skin Equivalent

**Figure 4:**
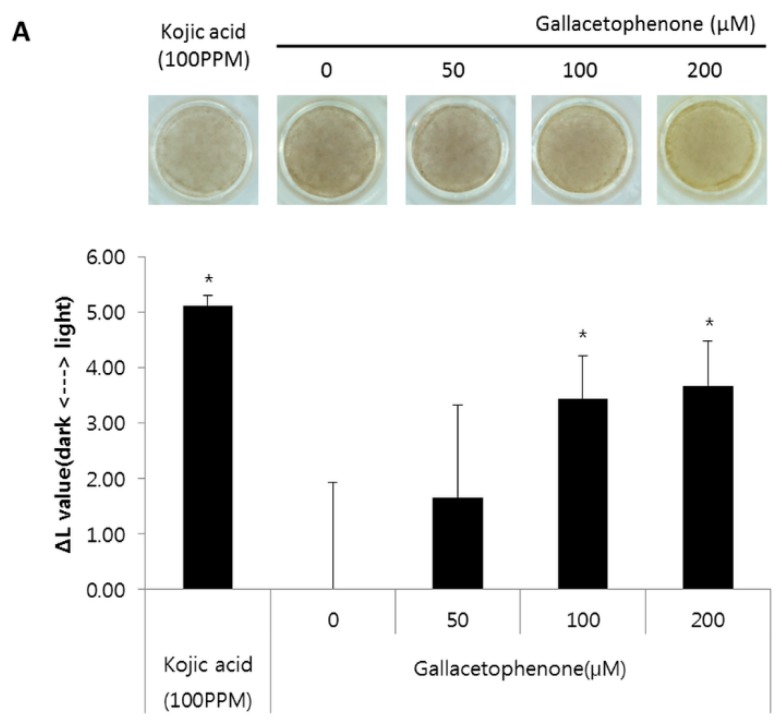

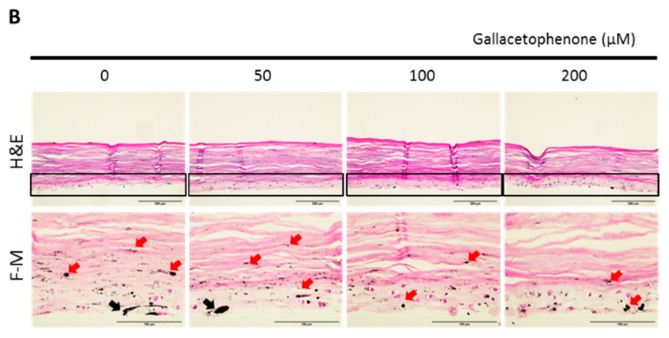
Optical and histological examination of whitening effects of gallacetophenone on 3D human skin equivalent. (**A**) Image of human skin equivalents; epidermal pigmentation level in skin equivalent was calculated by comparing variations in L* values (ΔL). Kojic acid was used as a positive control; (**B**) H&E (scale bar = 200 μm) and F-M staining (scale bar = 100 μm) were performed for histological examination. Black squares indicate transferred melanin. Data are expressed as the mean ± SD of at least three independent measurements (*p < 0.05).

To further demonstrate the skin lightening efficacy of gallacetophenone, we used the pigmented 3D human skin model, MelanoDerm. Although the most robust effect of reducing melanin content was observed in 1000 μM-treated melanocytes, a significant difference was observed in 30 μM-treated melanocytes. To identify the lowest effective concentration in human skin, treatment with gallacetophenone was started from 50 μM. As described in the Materials and Methods, MelanoDerm was exposed to 50, 100, and 200 μM of gallacetophenone-containing media for 14 days. After treatment, epidermal pigmentation was examined by optical and histological analyses. The gallacetophenone-treated 3D human skin equivalent showed a significant skin-whitening effect at >100 μM (Figure 4A). As shown in Figure 4A, a yellowish color was observed under treatment with 200 μM gallacetophenone. This yellowish color may have been derived from the color of gallacetophenone as it was treated for 2 weeks, which is two times longer than the treatment duration in the melanocyte assay. The images were analyzed by the L*, a, b system, were the L* value represents the relative brightness, the a value represents the balance between green and red, and the b value represents the balance between blue and yellow. Although the color appeared to be yellowish in 200 μM gallacetophenone-treated skin (in other words, the b value was much higher than the others), it did not affect the L* value. In addition, hematoxylin and eosin (H&E) staining and fontana-masson (F-M) staining were performed; as shown in Figure 4B, gallacetophenone did not induce significant cell and tissue toxicity, but melanin content was reduced in the gallacetophenone-treated 3D human skin equivalent. The part of the black square in the H&E image was stained with F-M. The F-M staining results showed that gallacetophenone decreased the number of active melanocytes (as indicated by black arrows) and melanins (as indicated by red arrows). Additionally, transfer of the produced melanin was inhibited in a dose-dependent manner; thus, the melanin content of the gallacetophenone-treated skin was lower than that of the non-treated one. Thus, we confirmed the whitening effect of gallacetophenone via the inhibition of melanin synthesis.

### 2.5. Gallacetophenone Affected the Expression of Melanogenic Proteins by Promoting Proteasomal-Mediated Degradation of Tyrosinase in Human Epidermal Melanocytes

**Figure 5:**
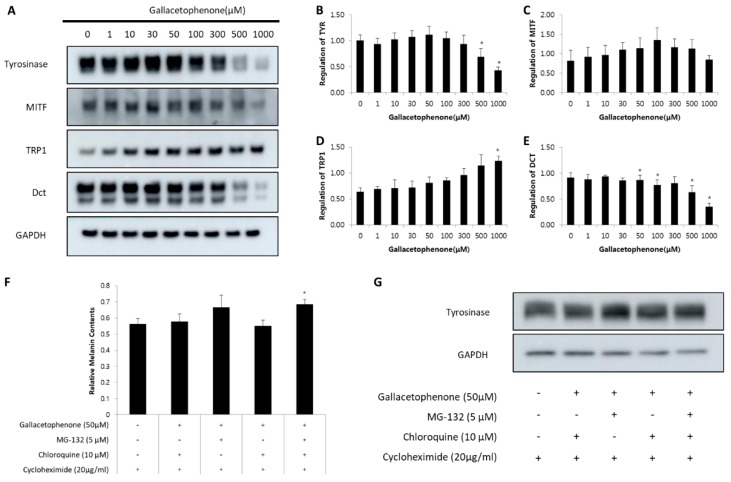
The expression level of melanogenic proteins and results of proteosomal inhibition assay. (**A**,**B**) Human epidermal melanocytes were treated with gallacetophenone in a dose-dependent manner. Signal intensity in the Western blot assay (**A**) was quantified using ImageJ software (National Institutes of Health, Bethesda, MD, USA) (**B**–**E**). To confirm the mechanism of gallacetophenone, the proteosomal inhibition assay was performed. The melanin contents were evaluated with proteosomal inhibitor MG-132 and lysosomal inhibitor chloroquine (**F**). The expression level of tyrosinase is shown in (**G**). Data are expressed as the mean ± SD of at least three independent measurements (*p < 0.05).

To elucidate the anti-melanogenic mechanism of gallacetophenone, we tested the regulation of the expression level of melanogenic enzymes. Human epidermal melanocytes were treated with various concentrations of gallacetophenone, as in other experiments, and the protein levels of tyrosinase, MITF, TRP1, and DCT were determined by Western blot assay. As shown in Figure 5, the levels of tyrosinase and DCT were reduced in a dose-dependent manner. However, MITF did not show any significant change. 

Although the expression level of tyrosinase was reduced by gallacetophenone, only the two highest concentrations significantly affected the protein production, with the melanin content being reduced at 30 μM gallacetophenone. This is possibly due to the fact that synthesis of new protein was not blocked as gallacetophenone might be involved in the degradation of tyrosinase. Although gallacetophenone might promote the degradation of tyrosinase, a new tyrosinase might be synthesized at the same time of degradation. Combining these two results, the expression level of tyrosinase has been shown in Figure 5A.

To confirm this, we evaluated tyrosine protein levels in human epidermal melanocytes treated with MG-132, a proteasome inhibitor, and/or chloroquine, a lysosomal proteolysis inhibitor. In these experiments, human epidermal melanocytes were treated with cycloheximide for 1 h to inhibit new protein synthesis. MG-132 and/or chloroquine were added, and cells were incubated for 1 h before incubating with gallacetophenone for 24 h. After treatment, melanin content and the expression level of tyrosinase were analyzed. Gallacetophenone-induced decrease in melanin content (Figure 5F) and tyrosinase was prevented (Figure 5G) by pretreatment with MG-132. Although the time for gallacetophenone treatment was too short relative to that in Figure 5A, the same tendency was observed. Collectively, these results indicate that gallacetophenone suppresses tyrosinase protein levels by promoting proteasomal-mediated degradation of tyrosinase.

### 2.6. Binding Mode of Gallacetophenone in the Active Site of Human Tyrosinase Model

**Figure 6:**
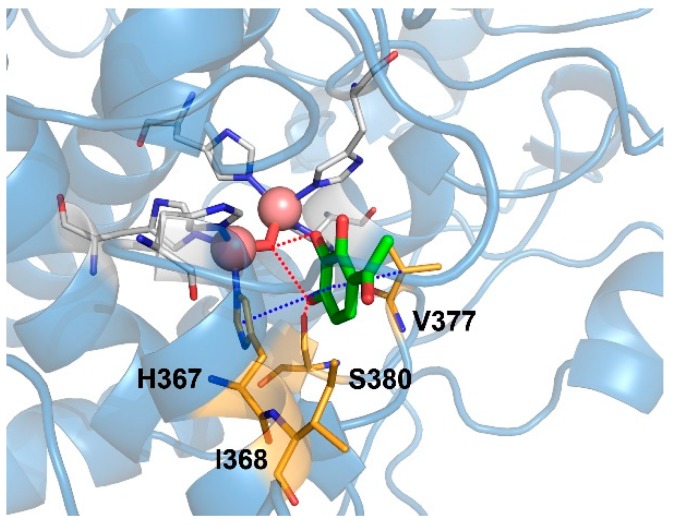
Proposed binding mode of gallacetophenone to the human tyrosinase model. The human tyrosinase model is represented in sky-blue color ribbons, and a water molecule (red stick) and histidine residues (white sticks) are shown chelated to copper ions (salmon spheres). The key interactions between gallacetophenone (green stick) and residues (bright orange stick) are represented as dotted blue lines (π–π stacking and π–alkyl interactions), and red line shows hydrogen bonding.

The binding mode of gallacetophenone in the active site of human tyrosinase model was stabilized by π–π stacking interactions of its phenyl ring with His367 and hydrophobic interactions with Ile368 and Val377, as shown in Figure 6. Particularly, gallacetophenone did not directly interact with the copper ions, but its hydroxyl groups made hydrogen bonds with a water molecule, bridging the copper ions within approximately 3.5 Å. In addition, gallacetophenone formed a hydrogen bond with the side chain of Ser380. The binding mode of gallacetophenone in our human tyrosinase model was the same as that of L-tyrosine, mimosine, and tropolone in human TRP1, consistent with the results obtained from the crystal structures of TRP1-3M mutant [29].

## 3. Discussion

Tyrosinase is the enzyme responsible for initiation of melanin synthesis and catalysis of the two steps of melanogenesis [22]. Many researchers, to date, have attempted to identify tyrosinase inhibitors with maximal efficacy but minimal side effects [32]. However, the major limitation has been that the 3D structure of human tyrosinase is not solved yet. Therefore, we conducted homology modeling to generate the 3D structure of human tyrosinase, and then performed docking-based high throughput virtual screening using 2770 compounds from an in-house natural compound library. The docking results were ranked and filtered based on docking score and ligand-efficiency score, and 74 compounds were finally selected by visual inspection. The binding mode of selected compounds in the active site of the human tyrosinase model involved some hydrophobic interactions with His367, Ile368, and Val377; hydrogen bonding with Ser380; and a water molecule bridging the copper ions. Of the 74 compounds, three hit compounds were selected by mushroom tyrosinase inhibitor screening assay. Although the assay was simple and fast in screening compounds, the difference between mushroom tyrosinase and human tyrosinase needs to be considered. Even within the same species, different sources of fungi are known to have different amino acid sequences of tyrosinase [33], and the tyrosinase-binding pocket of mushrooms and humans has already been reported to be different [34]. Although we considered this difference between human and mushroom by screening with a human tyrosinase model, it was not sufficient. Thus, in this study, we evaluated the anti-melanogenic effect of the selected hit compound, gallacetophenone, in human epidermal melanocytes and a 3D human skin equivalent. According to our findings, gallacetophenone inhibited melanin synthesis in human epidermal melanocytes (Figure 3) and exerted a depigmentation effect on 3D human skin equivalent (Figure 4). 

The selected gallacetophenone was extracted from *Rosa canina*, which belongs to the family Rosaceae and genus *Rosa* [35]. Although gallacetophenone itself has not yet been studied, *R. canina* pseudo-fruit has been traditionally used in preventive therapy and for the preparation of some foods such as jam, beverages, and probiotic drinks [36]. We therefore considered that gallacetophenone might be safe, as the original plant has been used even in food. Additionally, we determined in this study that gallacetophenone did not cause any toxicity at up to 1000 µM concentration in human epidermal melanocytes. Therefore, although the safety profiles over long-term exposure should be studied, we proposed that gallacetophenone has a potential as a natural anti-melanogenic compound.

We further evaluated the mechanism of gallacetophenone. The expression level of tyrosinase was evaluated. The expression level of tyrosinase reduced in a dose-dependent manner (Figure 5A), resulting in an anti-melanogenic effect. Additionally, we investigated the expression levels of other melanogenic proteins: MITF, TRP1, and TRP2/DCT; interestingly, only the expression level of TRP2/DCT was similarly reduced as that of tyrosinase. MITF, a major transcription factor regulating tyrosinase, showed no significant difference, and TRP1 increased slightly. These results suggested the possibility of gallacetophenone specifically inhibiting tyrosinase at the protein level, without affecting transcription.

Tyrosinase is degraded endogenously by proteasomes, which are multicatalytic proteinase complexes that selectively degrade intracellular ubiquitinated proteins. To determine whether proteasomal or lysosomal pathways are involved in mediating tyrosinase degradation by gallacetophenone, we evaluated tyrosine protein levels in human epidermal melanocytes. When we blocked proteasomal pathways, gallacetophenone-treated melanocytes did not reduce. All these results indicate that gallacetophenone suppresses tyrosinase protein levels by promoting proteasomal-mediated degradation of tyrosinase.

In conclusion, we demonstrated the inhibitory effect of gallacetophenone on melanogenesis and depigmentation effect in 3D human skin equivalent. This anti-melanogenic effect originated from the suppression of tyrosinase and promotion of its degradation, as suggested by the expression levels of melanogenic proteins in human epidermal melanocytes. We also demonstrated the binding of gallacetophenone to human tyrosinase, using computational molecular docking systems. Although functional validation is needed for long-term exposure, our findings revealed that gallacetophenone has potential as an effective and safe tyrosinase inhibitor, which could be a useful whitening ingredient in cosmetics [32]. 

## 4. Materials and Methods

### 4.1. Homology Modeling

To generate the 3D structure of human tyrosinase (hTYRO), we conducted homology modeling using Small-Molecule Drug Discovery Suite 2018-3 (Schrödinger, New York, NY, USA). The full sequence of hTYRO (P14679) was acquired from the UniProt database, and X-ray crystal structure of human tyrosinase-related protein 1 (TRP1, PDB ID: 5M8R) was used as a protein template, having 42.6% sequence identity with human tyrosinase. The co-crystallized ligand, copper ions instead of zinc ions, and a water molecule bridging the two metal ions were reflected in the human tyrosinase model structure. Protein Preparation Wizard (Schrödinger, NY, USA) was used to assign bond orders, charge states to ionizable residues, and to perform a restrained minimization of human tyrosinase model structure.

### 4.2. Docking-Based High Throughput Virtual Screening

The low-energy 3D structures of compounds in the in-house natural compound library were generated by LigPrep and docked into the active site of human tyrosinase model structure using Glide in the Standard Precision mode based on a grid box of 20 × 20 × 20 Å^3^ centered on the co-crystallized ligand. The protein–ligand interactions were analyzed by Discovery Studio Modeling Environment v4.02 (BIOVIA, San Diego, CA, USA). Thirty-five compounds with no binding mode were filtered out. The docking results of 2735 compounds were ranked and filtered by docking score and ligand-efficiency score, and finally 74 compounds satisfying the conditions of hydrophobic interactions with His367, Ile368, and Val377; hydrogen bonds with Ser380; and a water molecule bridging the copper ions were selected by visual inspection.

### 4.3. Reagents and Antibodies

Gallacetophenone was purchased from Biosynth Carbosynth (Berkshire, UK), isolindleyin was purchased from ChemFaces (Wuhan, China), and ethyl caffeate was purchased from ALB Technology Limited (Kowloon, Hong Kong, China). Methyl 3,4,5-trihydroxybenzoate, Ethyl 3,4,5-trihydroxybenzoate, KA, cycloheximide, MG-132, and chloroquine were purchased from Sigma-Aldrich (St. Louis, MO, USA). Antibodies against tyrosinase, TRP1, and TRP2 were purchased from Santa Cruz Biotechnology (Dallas, TX, USA). Antibody against MITF was purchased from Neomarkers (Carlsbad, CA, USA), and GAPDH was obtained from Cell Signaling Technology (Danvers, MA, USA).

### 4.4. Cell Culture and Viability Assay

Moderately pigmented human epidermal melanocytes (HEMn-MP) were purchased from Cascade Biologics (Portland, OR, USA) and cultured in Medium 254 (#M254500) supplemented with Human Melanocyte Growth Supplement (Life Technologies, Carlsbad, CA, USA). Cells were incubated at 37 °C containing 5% CO_2_. Cells from passages three to six were used for subsequent experiments. Cell viability was tested using a Cell Counting Kit-8 (CCK-8) according to manufacturer’s instruction (Dojindo, Tokyo, Japan). 

### 4.5. Mushroom Tyrosinase Inhibitor Screening Assay

A tyrosinase inhibitor assay kit was purchased from Abcam (Cambridge, UK). We investigated the mushroom tyrosinase inhibition rate according to the manufacturer’s instructions. Briefly, indicated concentrations of gallacetophenone, ethyl caffeate, and isolindleyin were mixed with enzyme solution and incubated at 25 °C for 10 min. The substrate solution was added, and absorbance was measured at 510 nm in a kinetic mode for 60 min at 25 °C. Relative inhibition rate of mushroom tyrosinase was calculated according to the manufacturer’s instructions. Arbutin, a well-known tyrosinase inhibitor, was used as a positive control.

### 4.6. Melanin Assay

Human epidermal melanocytes were seeded in 6-well plates and incubated for 24 h. They were treated with various concentrations of gallacetophenone for 7 days. Media containing the indicated concentrations of gallacetophenone were changed every 2 or 3 days. All cells were washed with phosphate-buffered saline (PBS) and dissolved in 1N NaOH at 60 °C for 1 h. Cell lysates were transferred to a 96-well plate, and absorbance was measured at 405 nm. The values were normalized based on the protein concentrations in each sample well.

### 4.7. Western Blotting

Cells were gently washed twice with cold PBS and extracted by incubation in RIPA lysis buffer (Cell Signalng Technology, ‎Danvers, MA, USA) containing protease inhibitors (Calbiochem, La Jolla, CA, USA) for 30 min at 4 °C. The cell lysates were centrifuged at 13,000 rpm for 30 min. Total protein concentrations were measured using a BCA assay kit (Sigma-Aldrich, St. Louis, MO, USA), and 15 μg of total protein was separated by SDS-polyacrylamide gel electrophoresis on 4–12% gradient Bis-Tris gels (Thermo Fisher Scientific, Waltham, MA, USA). The proteins were transferred to nitrocellulose membranes (Thermo Fisher Scientific, Waltham, MA, USA), which were then blocked in 10% blocking solution and probed with primary antibodies at 4 °C overnight. It was washed with Tris-buffered saline containing 0.2% Tween-20 (TBST) and exposed to secondary antibodies for 1 h at room temperature. Membranes were rinsed thrice with TBST. Chemiluminescent signal was developed using ECL Western blotting reagent (GE Healthcare, Hatfield, UK). The signal intensity was quantified using ImageJ software (National Institutes of Health, Bethesda, MD, USA) and normalized to that of GAPDH. 

### 4.8. Three-Dimensional (3D) Human Skin Equivalent

MelanoDerm (MEL-300-B; MatTek Corp., Ashland, MA, USA) was used for lightening skin in a human skin equivalent model. MelanoDerm was grown at 37 °C in a humidified 5% CO_2_ incubator using EPI-100-NMM-113-PRF medium (MatTek Corp., Ashland, MA, USA). Different concentrations of gallacetophenone were added to the culture medium every alternate day for 14 days. Thereafter, optical and histological analyses were performed for examining epidermal pigmentation. Epidermal pigmentation level in human skin equivalent was calculated by comparing variations in L* values (a lightness/darkness index) on days 1 and 14 and estimating the difference between them (ΔL). Histological examination using H&E and F-M staining was eventually performed to confirm the results. 

### 4.9. Statistical Analysis

All data are presented as the mean ± SD (standard deviations). Statistical significance was determined by Student’s *t*-test. A *p*-value < 0.05 was considered to be statistically significant.

## 5. Patents

A patent has been filed for gallacetophenone as a whitening compound. [Patent Application Number: 10-2020-0030898].

## Supplementary Materials

Supplementary materials can be found at https://www.mdpi.com/1422-0067/21/9/3144/s1. Figure S1: A total of 74 compounds, from docking-based virtual screening, were tested in mushroom tyrosinase inhibitor screening assay. Figure S2: Structures of the mushroom tyrosinase inhibitory compounds.

## Abbreviations

hTYRO	human Tyrosinase
MITF	Microphthalmia-associated transcription factor
TRP1	Tyrosinase-related protein 1
TRP2/DCT	Tyrosinase-related protein 2 or dopachrome tautomerase
DOPA	Dihydroxyphenylalanine
GAPDH	Glyceraldehyde-3-phosphate dehydrogenase
H&E	Hematoxylin and eosin
F-M	Fontana-Masson staining
