# Supplementary material for: Tyrosinase-Targeting Gallacetophenone Inhibits Melanogenesis in Melanocytes and Human Skin- Equivalents
Source: Int J Mol Sci. 2020 Apr 29;21(9):3144. doi: 10.3390/ijms21093144 (PMC7246559; doi:10.3390/ijms21093144)
Supplement: Supplementary file 1 [file ijms-21-03144-s001.zip › Supplementary Files.docx]

Supplementary Materials


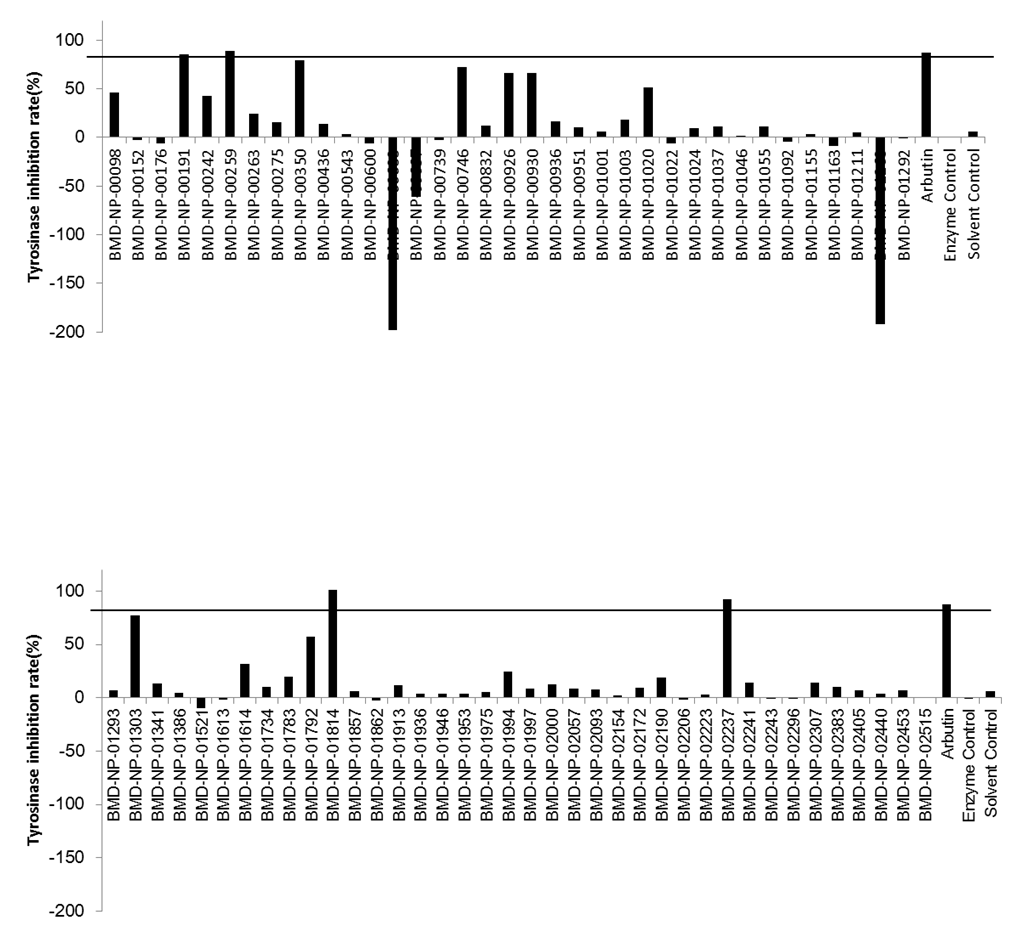


**Figure S1.** A total of 74 compounds, from docking-based virtual screening, were tested in mushroom tyrosinase inhibitor screening assay. 5mM of all compounds were treated to mushroom tyrosinase. Black line on the graph indicated 80% inhibition rate. Only four compounds, BMD-NP-00191 (Isolindleyin), BMD-NP-00259 (Ethyl caffeate), BMD-NP-01814 (Glabridin), and BMD-NP-02237 (Gallacetophenone) showed > 80% inhibition rate, including arbutin as the positive control.


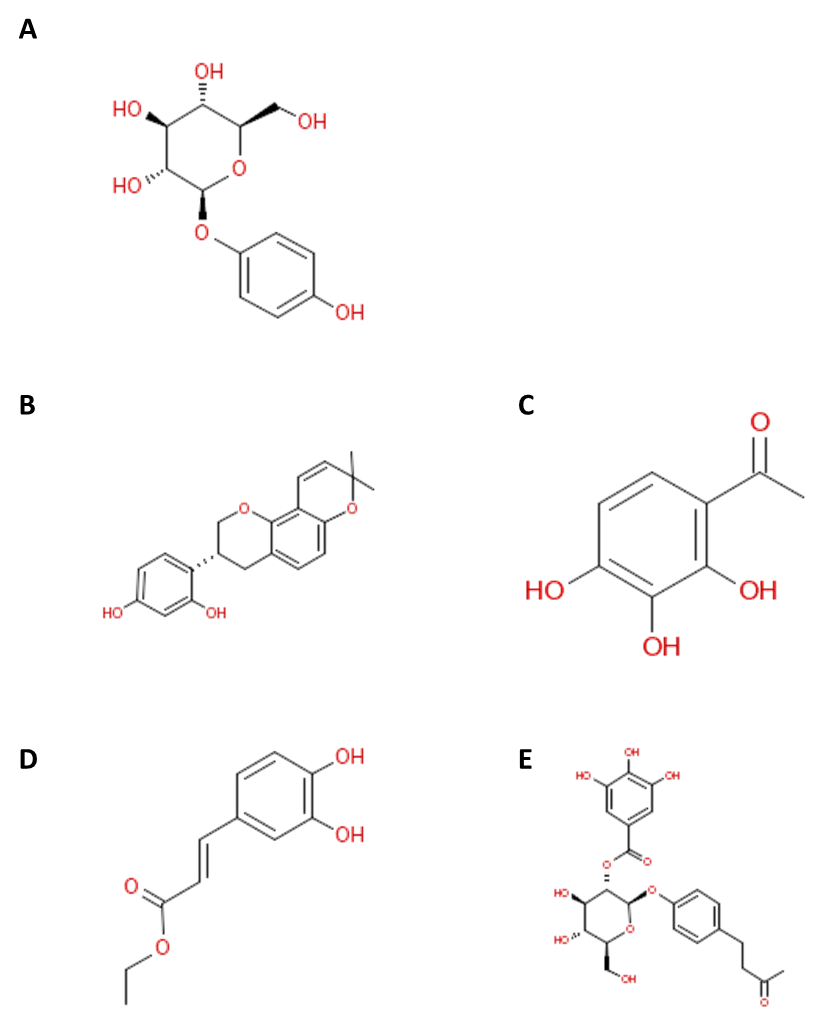


**Figure S2.** Structures of the mushroom tyrosinase inhibitory compounds. (**A**) Arbutin as a positive control with 87% inhibition rate; (**B**) BMD-NP-01814 (Glabridin) with 101% inhibition rate; (**C**) BMD-NP-02237 (Gallacetophenone) with 93% inhibition rate; (D) BMD-NP-00259 (Ethyl caffeate) with 89% inhibition rate; (E) BMD-NP-00191 (Isolindleyin) with 85% inhibition rate.
